# Computational Pathology to Discriminate Benign from Malignant Intraductal Proliferations of the Breast

**DOI:** 10.1371/journal.pone.0114885

**Published:** 2014-12-09

**Authors:** Fei Dong, Humayun Irshad, Eun-Yeong Oh, Melinda F. Lerwill, Elena F. Brachtel, Nicholas C. Jones, Nicholas W. Knoblauch, Laleh Montaser-Kouhsari, Nicole B. Johnson, Luigi K. F. Rao, Beverly Faulkner-Jones, David C. Wilbur, Stuart J. Schnitt, Andrew H. Beck

**Affiliations:** 1 Department of Pathology, Massachusetts General Hospital, Harvard Medical School, Boston, Massachusetts, United States of America; 2 Department of Pathology, Brigham and Women's Hospital, Harvard Medical School, Boston, Massachusetts, United States of America; 3 Department of Pathology, Beth Israel Deaconess Medical Center, Harvard Medical School, Boston, Massachusetts, United States of America; University of Torino, Italy

## Abstract

The categorization of intraductal proliferative lesions of the breast based on routine light microscopic examination of histopathologic sections is in many cases challenging, even for experienced pathologists. The development of computational tools to aid pathologists in the characterization of these lesions would have great diagnostic and clinical value. As a first step to address this issue, we evaluated the ability of computational image analysis to accurately classify DCIS and UDH and to stratify nuclear grade within DCIS. Using 116 breast biopsies diagnosed as DCIS or UDH from the Massachusetts General Hospital (MGH), we developed a computational method to extract 392 features corresponding to the mean and standard deviation in nuclear size and shape, intensity, and texture across 8 color channels. We used L1-regularized logistic regression to build classification models to discriminate DCIS from UDH. The top-performing model contained 22 active features and achieved an AUC of 0.95 in cross-validation on the MGH data-set. We applied this model to an external validation set of 51 breast biopsies diagnosed as DCIS or UDH from the Beth Israel Deaconess Medical Center, and the model achieved an AUC of 0.86. The top-performing model contained active features from all color-spaces and from the three classes of features (morphology, intensity, and texture), suggesting the value of each for prediction. We built models to stratify grade within DCIS and obtained strong performance for stratifying low nuclear grade vs. high nuclear grade DCIS (AUC = 0.98 in cross-validation) with only moderate performance for discriminating low nuclear grade vs. intermediate nuclear grade and intermediate nuclear grade vs. high nuclear grade DCIS (AUC = 0.83 and 0.69, respectively). These data show that computational pathology models can robustly discriminate benign from malignant intraductal proliferative lesions of the breast and may aid pathologists in the diagnosis and classification of these lesions.

## Introduction

The pathological classification of ductal carcinoma in situ (DCIS) versus usual ductal hyperplasia (UDH) on core biopsy of has major implications for patient management. UDH is considered a benign proliferation, and patients with UDH carry only a small increased risk of developing subsequent breast cancer compared with patients without proliferative breast disease [1]. No treatment is necessary, and clinical management includes the continuation of routine breast cancer screening. In contrast, DCIS is a preinvasive malignant proliferation, and approximately 25% of patients diagnosed with DCIS on core biopsy are found to have invasive carcinoma upon surgical excision [2]. Primary treatment recommendations for DCIS include lumpectomy with or without whole breast radiation therapy and/or postoperative tamoxifen or total mastectomy with or without sentinel lymph node biopsy [3]. Thus, DCIS patients receive aggressive treatment, while UDH patients receive no treatment.

The pathological distinction of DCIS and UDH is based on multiple architectural and cytologic features, with nuclear atypia being particularly important in distinguishing the benign (UDH) from the malignant (DCIS) lesions. Although the defining features of DCIS and UDH are well established, the accurate and reproducible categorization of intraductal proliferative breast lesions into these categories remains a challenge in many cases, even for experienced pathologists [4]–[10]. The lack of reproducible and objective methods for classifying intraductal proliferative lesions of the breast has clear negative consequences, potentially resulting in both over- and under-treatment in clinical practice and complicating the use of pathological diagnoses to guide research in this area.

Given the importance of accurate and reproducible pathological diagnosis for guiding clinical care and translational research, there would be value to the development of computational tools to aid in the morphological characterization of intraductal proliferative lesions. Descriptive morphological features of nuclear atypia may be modeled with digitization of H and E-stained sections followed by image processing and analysis. The process involves image capture via digital photography or whole slide scanning [11], image segmentation via manual or automated methods [12], the measurement of extracted features, and the application of statistical methods to determine the association of features and feature-based predictive models with pathological diagnosis or clinical outcome [13], [14]. Quantitative nuclear features have been demonstrated to correlate with pathological findings in prior studies of mitotic activity [15], [16] and epithelial proliferations of the breast [17]–[21]. In particular, the measurement of nuclear area has been demonstrated to have diagnostic and prognostic value in malignant and premalignant lesions [22]–[27].

Although the current literature demonstrates correlations between quantitative features and proliferative breast lesions, this methodology has not yet been widely adopted within the pathology community. One reason is due to the inconvenience of applying previously published quantitative methods, which historically have required significant manual intervention (e.g. for nuclear tracing) prior to the measurement and extraction of one-to-several quantitative features of cellular morphology. A second reason is few prior studies have used external validation datasets, limiting our knowledge of the generalizability and robustness of the findings. A third major challenge has been that few pathology laboratories have possessed the requisite hardware and software for implementing computational pathology algorithms, and thus, it has been challenging to translate research advances to clinical practice. However, increasing numbers of laboratories are acquiring whole slide imaging (WSI) platforms and digital pathology tools, which should significantly facilitate the dissemination and ultimate clinical translation of computational pathology algorithms [11], [14], [28], [29].

To build on the strengths and to address some of the limitations of prior work in this area, we designed and implemented a computational pathology method for the identification of nuclei and the quantification of nuclear features from histological images of intraductal proliferative lesions of the breast. In this study, we demonstrate the ability of this method to build classification models to discriminate DCIS from UDH and to discriminate low grade from high grade DCIS. These investigational tools provide new biological insights into the key cellular phenotypic differences that differentiate UDH and DCIS and ultimately, with further development, may provide real-time decision support to aid pathologists in the interpretation of proliferative lesions of the breast. All image processing code, images, and statistical code are provided at the accompanying website: earlybreast.becklab.org.

## Materials and Methods

### Patient samples

#### Massachusetts General Hospital (MGH) Dataset

The study was approved by the Partners Human Research Committee (Partners IRB), and the Partners IRB waived the need for consent. Cases were identified via a search of breast biopsies with a diagnosis of DCIS or UDH at MGH. 80 cases of DCIS and 36 cases of UDH from MGH were included in the study.

Core biopsy tissue was processed according to standardized laboratory protocol. Formalin fixed and paraffin embedded (FFPE) tissue was cut into 5 µm sections and stained with hematoxylin and eosin. The pathological grading of DCIS cases were obtained from the diagnostic pathology reports, and cases of DCIS were graded as low, intermediate or high based on the degree of nuclear atypia. If a case was reported to be intermediate between two grades, for the purposes of this analysis we classified the case as the lower of the grades.

#### Beth Israel Deaconess Medical Center (BIDMC) Dataset

The study was approved by the Beth Israel Deaconess Medical Center IRB, and the IRB waived the need for consent. Cases were identified via a search of breast biopsies with a diagnosis of DCIS or UDH at BIDMC. 20 cases of DCIS and 31 cases of UDH from BIDMC were included in the study. Similar to the MGH data-set, FFPE tissue was cut into 5 µm sections and stained with hematoxylin and eosin. The pathological grading of DCIS cases were obtained from the diagnostic pathology reports, and cases of DCIS were graded as low, intermediate or high based on the degree of nuclear atypia. If a case was reported to be intermediate between two grades, for the purposes of this analysis we classified the case as the lower of the grades.

### Image acquisition

One representative diagnostic hematoxylin and eosin stained slide per case was digitized using Philips Ultra Fast Scanner 1.6 (Philips Digital Pathology; Best, Netherlands) at 40× magnification with a resolution of 0.25 µm per pixel. Whole slide images were reviewed, and diagnostic ROIs (1 to 4 per case) were manually selected for image analysis. For cases with greater than 4 diagnostic foci, up to 4 regions with the highest cellularity were selected. The MGH cases were scanned using a Philips Scanner at the MGH facility, while the BIDMC cases were scanned using a Philips Scanner at BIDMC.

### Image processing and feature extraction

The proposed image processing and analysis framework consists of three main steps: *nuclei segmentation*, *nuclei feature computation* and *statistical analysis and machine learning* on computed features.

#### Nuclei segmentation

**Figure 1:**
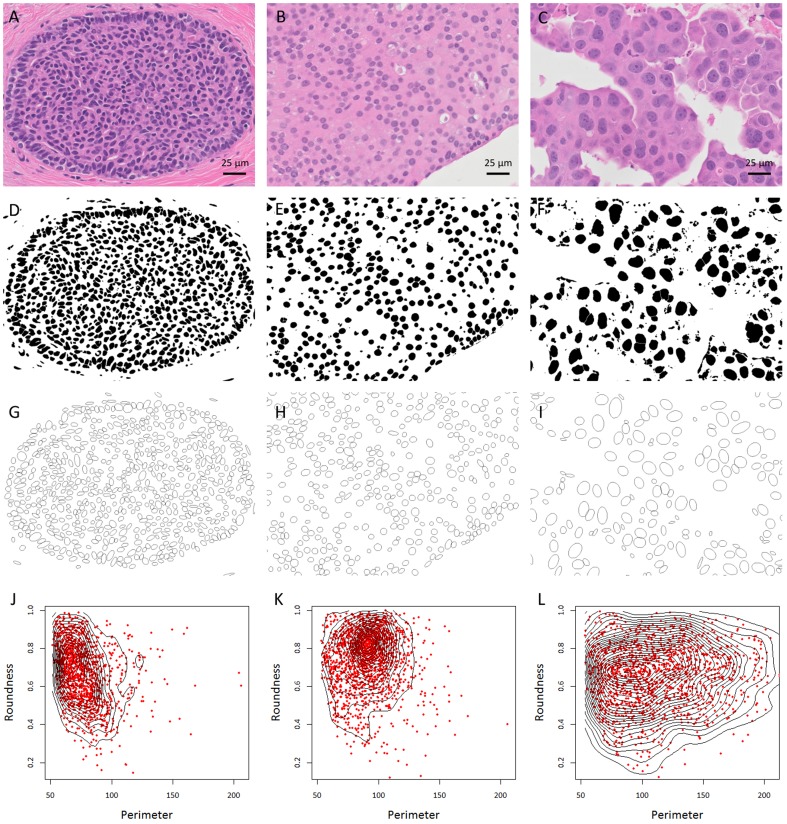
Image processing of UDH (A), low grade DCIS (B), and high grade DCIS (C) includes conversion of hematoxylin and eosin-stained images to binary images via automated threshold and watershed segmentation (D-F). Elliptical approximations of identified nuclear objects are shown (G-I). Quantitative measurements (perimeter and circularity in this example) of nuclear distributions, where each data point represents one nuclear object, are shown as scatterplots and contour plots of two-dimensional kernel density estimations for each case (J-L).

Nuclei segmentation was performed using Fiji (ImageJ, National Institutes of Health) [30]. For each image, the segmentation algorithm was applied for nuclei segmentation. Initially, the RGB color image was converted into HSV color space, in which image intensity (luma) is separated from color information (chroma), which makes the HSV more closely match human perception. Color thresholding was performed to obtain nuclear regions which were later processed with morphological operations to fill holes and merge scattered nuclear regions. Touching and overlapping nuclei were separated by watershed transformation. Following nuclear segmentation, a size filter of 200–4000 pixels was applied to exclude extracted objects of extremely small or large size to improve the specificity of nuclear detection. Identified objects were then analyzed from the original image to measure quantitative features of morphology and color of each nucleus (Fig. 1).

#### Nuclei feature computation

After nuclei segmentation, we computed morphological and statistical features from the selected nuclear regions. The computed morphological features include shape and geometric features, which are: area, perimeter, equivalent spherical perimeter, bounding rectangle (width and height), fit ellipse (major and minor axis), shape descriptors (circularity, aspect ratio, roundness and solidity) and Feret's diameter.

The statistical features are intensity based (first order) and texture based (second order). We also explored the statistical features in different color models. In order to study the specific information carried by the hematoxylin stain, which highlights different cellular structures in the tissue, we separated hematoxylin and eosin stains using color deconvolution [31]. Color deconvolution reduced the problem of color variations in tissue appearance due to variation in tissue preparation, stain reactivity from different batches, protocol and scanners. In addition, different color models are proposed to separate a color into more useful components that may bring new information to the system. In this framework, our goal is to investigate the various color channels of different color models and select those channels that produce the top-performing classification models. We convert RGB images into two other color models, namely HSV (more intuitive for human perception) and Lab and Luv (uniform color separation).

In H&E stained images, nuclear and cytoplasmic regions appear as hues of blue and purple while extracellular material have hues of pink. In order to reduce the influence of extracellular region intensity, the RGB images are transformed into a new image called Blue-Ratio (BR) image to accentuate the nuclear dye [32] as:
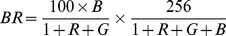
where R, G and B are red, green and blue channels of RGB, respectively. In a BR image, a pixel with a high blue intensity relatively to its red and green components is given a high value, whereas, a pixel with a low blue intensity as compared to its red and green components is given a low value. As we are interested in nuclei, which appear as blue-purple areas, Blue ratio intensity indicates spatial distribution of nuclear content in the image. For statistical feature computations, we selected eight color channels; red, green and blue from RGB color model, lightness (Value) from HSV color model, lightness from Lab and Luv color model, BR grey scale image and Hematoxylin channel from H&E color deconvolution.

The first order statistical features determine the distribution of grey level values within the nuclei regions. Using grey level information of the selected color channels, we computed mean, median, standard deviation, skewness and kurtosis. These five features were computed for each nucleus in selected eight color channels, resulting in a total of 40 first order statistical features.

We computed two types of second order statistical features using grey level Haralick co-occurrence [33] and run-length matrices [34]. The co-occurrence matrix *GLCM (i,j; d,ϑ)* is square with dimension *N_g_* where *N_g_* is the total number of grey levels in the image. The value at *i* ^th^ and *j* ^th^ column in the matrix is produced by counting the total occasions a pixel with value *i* is adjacent to a pixel with value *j* at a distance *d* and angle *ϑ*. Then the whole matrix is divided by the total number of such comparisons that have been made. Alternatively we can say that each element of *GLCM* matrix is considered as the probability that a pixel with grey level *i* is to be found with pixel with grey level *j* at a distance *d* and angle *ϑ*. We defined adjacency in four directions (vertical, horizontal, left and right diagonals) with one displacement vector, which produced four *GLCMs* matrices. In our case, texture information is rotationally invariant. So, we take the average in all four directions and produce one *GLCM* matrix. Later, we compute 8 features proposed by Haralick from the *GLCM* in order to identify texture more compactly. These eight features are correlation, cluster shade, cluster prominence, energy, entropy, hara-correlation, homogeneity and inertia. These eight features were computed for each nucleus in the selected eight color channels, resulting in a total of 64 co-occurrence features.

The set of consecutive pixels, with the same grey level, collinear in a given direction, constitute a grey level run length matrix *GLRLM (i,j; ϑ)*. The dimension of *GLRLM* is *N_g_×R*, where *N_g_* is the number of grey levels and *R* is the maximum run length. Similar to the GLCM, we compute *GLRLMs* for four directions and later average them. The 10 run-length features, derived from *GLRLM*, are short run emphasis (SRE), long run emphasis (LRE), grey-level non-uniformity (GLN), run length non-uniformity (RLN), low grey level runs emphasis (LGLRE), high grey level runs emphasis (HGLRE), short run low grey level emphasis (SRLGLE), short run high grey level emphasis (SRHGLE), long run low grey level emphasis (LRLGLE) and long run high grey level emphasis (LRHGLE). These features were computed for each nucleus in the selected eight color channels, resulting in a total of 80 run-length features. In total, we computed 196 texture features for each nucleus.

Prior to statistical and machine learning-based analyses, feature measurements were summarized at the patient level by computing the mean and standard deviation of each feature per patient, producing a total of 392 summary features per patient.

*Statistical analysis and machine learning*: We performed logistic regression with Lasso regularization[35] to build multivariate image feature-based models to classify DCIS versus UDH and low grade versus high grade DCIS. The analyses were implemented in R (http://www.r-project.org/), using the *glmnet* [36] package. Lasso regularization was used to create simpler models, less prone to overfitting, than those that would be obtained from standard logistic regression. The Lasso procedure consists of performing logistic regression with an L_1_ regularization penalty, which has the effect of shrinking the regression weights of the least predictive features to 0 [35]. The amount of the penalty (and the number of non-zero features in the model) is determined by the regularization parameter λ. This method has been shown to perform well in the setting of colinearity[37] and has been widely used to build predictive models from high-dimensional data in translational cancer research [38]–[40]. Features were standardized separately in the MGH and BIDMC data-sets prior to model construction, using the default setting in *glmnet*. We evaluated model performance within the MGH data-set by 9-fold cross-validation. For external validation on the BIDMC cases, we selected the value of λ that achieved the maximum AUC in cross-validation on the MGH data-set and applied this fixed model to the BIDMC cases. Model performance was assessed by computing the AUC.

### Accompanying website

All images, image processing code, statistical code, and results are provided at the accompanying website: http://earlybreast.becklab.org and data are deposited at the Dryad database (http://dx.doi.org/10.5061/dryad.pv85m).

## Results

### Construction and evaluation of a computational pathology model to discriminate DCIS from UDH

#### DCIS and UDH Analyses in the Massachusetts General Hospital Dataset

**Figure 2:**
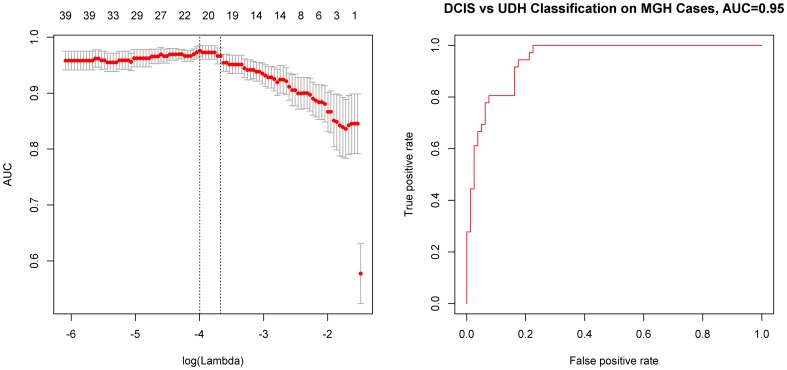
Learning a logistic regression model to distinguish DCIS vs. UDH on the MGH Dataset. A. Area under the receiver operating characteristic curve (AUC) based on predictions made on held-out cases in cross-validation with respect to log of the regularization parameter λ (bottom) and the number of features active in the model (top). Dotted lines indicate λ of model with maximum AUC (left) and largest λ such that the AUC is within one standard error of the maximum (right). B. Receiver operating characteristic (ROC) curve based on predictions made in cross-validation for the λ value that maximized the AUC in cross-validation for discriminating DCIS vs UDH on the MGH dataset.

We performed L_1_-regularized logistic regression to construct classification models to discriminate DCIS (n = 80) from UDH (n = 36). The top-performing model contained 22 active features (**Table 1**) and achieved an AUC of 0.95 in cross-validation (**Fig. 2**).

**Table 1:** Features and weights in the DCIS vs. UDH classification model.

Feature Name	Summary Function	Feature Class	Color Space	Weight
Mean_Variance_Red	Mean	Intensity	Red (RGB)	0.000875
Mean_Kurtosis_Green	Mean	Intensity	Green (RGB)	−3.15038
Mean_Kurtosis_BR	Mean	Intensity	BlueRatio	0.002031
Mean_Skewness_BR	Mean	Intensity	BlueRatio	0.523952
SD_Mean_Red	SD	Intensity	Red (RGB)	−0.05881
SD_Variance_Green	SD	Intensity	Green (RGB)	0.000543
Mean_Minor	Mean	Morphology	N/A	−0.13152
Mean_Round	Mean	Morphology	N/A	−28.1278
Mean_IDM_Blue	Mean	Texture	Blue (RGB)	101.3997
Mean_ClusterShade_HSV	Mean	Texture	V (HSV)	0.000004
Mean_GLN_Lab	Mean	Texture	L (Lab)	−0.074964
Mean_LGLRE_Lab	Mean	Texture	L (Lab)	−5239.083
Mean_SRLGLE_Lab	Mean	Texture	L (Lab)	0.000001
Mean_LRLGLE_Lab	Mean	Texture	L (Lab)	−329.1062
Mean_GLN_Luv	Mean	Texture	L (Luv)	−0.000184
Mean_LGLRE_Luv	Mean	Texture	L (Luv)	−0.058987
Mean_SRLGLE_Luv	Mean	Texture	L (Luv)	−35.01663
Mean_LRLGLE_Luv	Mean	Texture	L (Luv)	−0.563487
Mean_GLN_HE	Mean	Texture	H (HE)	−0.592543
SD_Inertia_Red	SD	Texture	Red (RGB)	0.000525
SD_Entropy_Blue	SD	Texture	Blue (RGB)	−7.797402
SD_IDM_HSV	SD	Texture	V (HSV)	−89.83901

#### External Validation of the DCIS/UDH Classification Model

**Figure 3:**
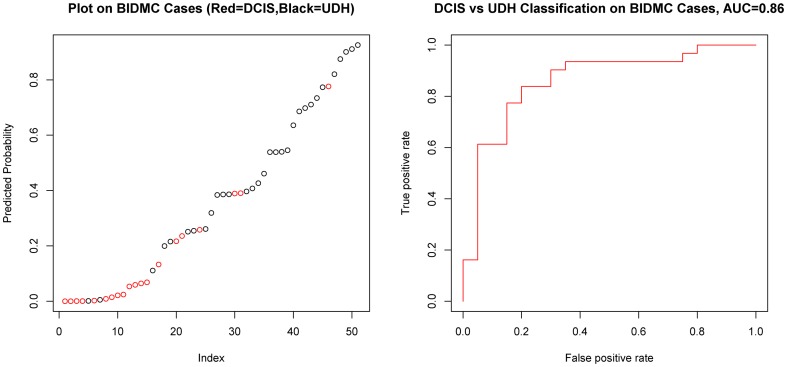
Validation of DCIS vs. UDH model on the BIDMC Dataset. A. Each point represents a case from the validation dataset. The cases are ranked based on the probability of UDH, which is indicated on the Y-Axis. The red points represent cases of DCIS and the black points represent cases of UDH. B. Receiver operating characteristic (ROC) curve based on based on predictions made on the BIDMC validation dataset for discriminating DCIS vs UDH.

**Figure 4:**
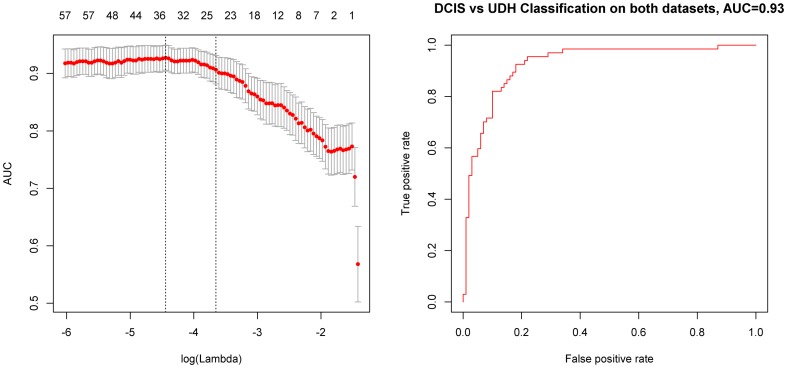
Learning a logistic regression model to distinguish DCIS vs. UDH on the MGH and BIDMC Datasets. A. Area under the receiver operating characteristic curve (AUC) based on predictions made on held-out cases in cross-validation with respect to log of the regularization parameter λ (bottom) and the number of features active in the model (top). B. Receiver operating characteristic (ROC) curve based on based on predictions made in cross-validation for the λ value that maximized the AUC in cross-validation for discriminating DCIS vs UDH on the MGH and BIDMC datasets.

The most important test of a predictive model is its ability to generalize to new, unseen data from an independent institution. Thus, we collected a set of 51 samples, containing cases of both DCIS (n = 20) and UDH (n = 31) as an external validation dataset. We processed the samples in a similar fashion to that described for the MGH samples. We trained the predictive model on the full MGH dataset and applied the fixed model to the BIDMC dataset. On the BIDMC dataset, the model showed strong classification performance, achieving an AUC = 0.86 (**Fig. 3**). Later, we combined both training and validation datasets and performed cross validation on all 167 cases, resulting in a model performance of AUC = 0.93 (**Fig. 4**).

We next compared the performance of the DCIS vs UDH classifier on specific grades of DCIS (**Table 2**) on the combined MGH and BIDMC datasets. The resulting dataset contains UDH (n = 67), low grade DCIS (n = 29), intermediate grade DCIS (n = 47) and high grade DCIS (n = 24) cases. We obtained strong performance for building classifiers to discriminate UDH vs. high grade DCIS (AUC = 0.97), UDH vs. low grade DCIS (AUC = 0.95), and UDH vs. intermediate grade DCIS (AUC = 0.90). These results show that the DCIS vs. UDH classifier performs well across all histologic grades of DCIS.

**Table 2:** Classification performance for DCIS and UDH classification models across a range of classification tasks and using varying subsets of features.

**Classification Performance, UDH vs. Grades of DCIS**			
	Total Features	Selected Features	AUC
UDH vs High Grade DCIS	392	26	97
UDH vs Low Grade DCIS	392	29	95
UDH vs Intermediate Grade DCIS	392	46	90
**Classification Performance, Between Grades of DCIS**			
	Total Features	Selected Features	AUC
Low Grade vs High Grade DCIS	392	17	98
Low Grade vs Intermediate Grade DCIS	392	8	83
Intermediate Grade vs High Grade DCIS	392	37	69
**Classification Performance, DCIS vs. UDH, with Different Subset of Features**			
	Total Features	Selected Features	AUC
Textural Features	288	27	91
Morphological Features	24	16	89
Intensity Features	80	24	85
**Classification Performance, DCIS vs. UDH, with Different Color Channels**			
	Total Features	Selected Features	AUC
Red Channel	46	28	90
V (HSV) Channel	46	26	89
L (Luv) Channel	46	23	88
L (Lab) Channel	46	23	88
BlueRatio Image	46	21	85
Green Channel	46	31	84
Blue Channel	46	28	84
H (H&E)	46	37	82

We next built models to discriminate between histologic grades of DCIS. We obtained strong performance for low-grade vs. high grade DCIS (AUC = 0.98), but only moderate performance for low grade vs. intermediate grade DCIS (AUC = 0.83) and for intermediate grade vs. high grade DCIS (AUC = 0.69). These results suggest the morphologic overlap between intermediate-and-low grade DCIS and between intermediate-and-high grade DCIS.

#### Analysis of Features in the Top-Performing DCIS vs. UDH Model

The top-performing DCIS vs. UDH model contained a total of 22 features, including intensity, morphology, and texture-based features (Table 1). Further, features from all color spaces contributed to the top-performing model, suggesting the added value of each.

#### Ablation analyses to compare performance of different feature classes and color spaces

To identify the subset of most informative features, we performed ablation analyses, in which we only consider a subset of features prior to model building and evaluation. Of the feature classes, we obtained the strongest performance with the texture features alone, which achieved an AUC of 0.91, followed by morphology and intensity features alone (AUC = 0.89 and 0.85, respectively). Of the color channels, the red channel features achieved the highest performance (AUC = 0.90), as compared with AUC = 0.89, 0.88 and 0.88 in the V (HSV), L (Lab) and L (Luv) channels, respectively. No subset of features achieved as strong performance as the full feature set (AUC = 0.93 in cross-validation), suggesting the added value of each class of feature and color-space.

## Discussion

Previous studies have used quantitative image analysis to aid in the histological diagnosis and grading of breast lesions. Nuclear size and shape have been associated with pathological findings such as tumor grade and necrosis in malignant cytological and surgical breast specimens [17]–[20]. Morphometric nuclear size measurements, including area, diameter, and short axis, have also been associated with survival in patients with invasive carcinoma [22], [23]. A comprehensive machine learning-based analysis of invasive breast carcinoma morphology has identified both epithelial and stromal features associated with survival [38]. Two prior studies of male breast carcinoma have associated morphometric parameters with tumor grade, immunohistochemical staining of tumor-associated proteins, and clinical outcome [21], [24].

Although relatively less work has focused on the evaluation of breast biopsies with benign pathological diagnoses, nuclear morphometry in benign proliferative lesions has been associated with the subsequent development of breast cancer [25], [26]. These previous studies, with methods ranging from manual measurement to automated computational segmentation of cellular morphology, suggest the value of developing and applying methods in quantitative image analysis to link morphologic phenotypes with pathological diagnoses and clinical outcomes.

In contrast to most prior studies, we take a more unbiased data-driven approach, where we develop automated image processing software to extract a relatively high dimensional nuclear feature set (392 features) from each image and then use machine learning-based methods to build models to predict pathological diagnosis. Dundar *et al*. developed a morphometric pipeline to measure nuclear perimeter, aspect ratio and mean gray level intensity across 62 training cases and 33 test cases of UDH, DCIS, and atypical ductal hyperplasia (ADH), and they demonstrated an overall accuracy of 87.9% in distinguishing clinically actionable from non-actionable lesions [27]. This prior study and others have largely focused on the quantitation of existing diagnostic criteria used by surgical pathologists for disease classification. Our study builds on this approach and identifies novel morphological features that are important in discriminating pathological diagnoses of breast disease. Furthermore, our study demonstrates the ability for pathology image analysis to accurately classify proliferative breast lesions across two institutions using different histological protocols. This finding suggests that these techniques may be implemented across multiple laboratories.

The image processing method could be further developed in several areas. First, the current system requires expert selected regions of interest. The nuclear detection algorithm currently implemented will not perform well on images that are pure stroma and assumes that the image is selected to enrich for cells involved in a breast epithelial proliferative lesion. A second area for future technical development is incorporation of relational nuclear features (e.g. average distance between nuclei, proportion overlapping nuclei) and spatial nuclear features (e.g. nuclear streaming). A third area for future development is the inclusion of stromal features into the predictive model. It is well known that DCIS may develop reactive-type stroma, and this feature may have value in diagnosis and prognosis. Lastly, performance may be further improved through the use of additional machine learning methods, including convolutional neural networks, which have recently shown strong performance for a range of image classification tasks [41], [42].

Despite these limitations, the current study represents a first step in applying a computational pathology approach to the classification of intraductal proliferations of the breast. Our study demonstrates that models comprised of relatively small sets of quantitative nuclear features can achieve high accuracy for the classification of UDH vs. DCIS and low grade vs high grade DCIS. Building on this work, we plan to extend the method to the analysis of additional challenging intraductal proliferative lesions, including ADH, which represents a major challenge in diagnostic pathology[9] and is an area where a computational algorithm may be particularly useful for clinical practice.

With further development and validation, we envision this approach could be incorporated into clinical practice in several ways. Today, the standard method used to differentiate difficult cases on the borderline between low grade DCIS and UDH is to perform immunohistochemistry (IHC) for estrogen receptor (ER) and basal cytokeratins (CK5/6), with low grade DCIS tending to show the pattern of ER positive and CK5/6 negative, while UDH shows a mosaic-staining pattern for CK5/6 and variable ER positivity [43]. A computational pathology algorithm may replace the need for IHC altogether in a subset of cases, resulting in increased turnaround time and decreased cost, or alternatively, the algorithm may supplement the use of IHC for difficult cases, providing another layer of evidence to support one diagnosis or the other. In addition, we envision that a computational pathology algorithm for intraductal proliferative lesions could serve as a real-time second reader, which could point the pathologist to suspicious lesions or could trigger the pathologist's attention when the pathological diagnosis disagrees with the computational interpretation, and in this way, the algorithm could be used to identify cases requiring additional diagnostic work up.

The image processing algorithms, full set of images used in the analysis, and the code for statistical analysis are made publicly available on the accompanying website (earlybreast.becklab.org) and data are deposited at the Dryad Database (http://dx.doi.org/10.5061/dryad.pv85m).

## References

[1] Dupont WD, Page DL (1985) Risk factors for breast cancer in women with proliferative breast disease. N Engl J Med 312:146–151. 3965932 10.1056/NEJM198501173120303

[2] Brennan ME, Turner RM, Ciatto S, Marinovich ML, French JR, et al (2011) Ductal carcinoma in situ at core-needle biopsy: meta-analysis of underestimation and predictors of invasive breast cancer. Radiology 260:119–128 10.1148/radiol.11102368 21493791

[3] Kane RL, Virnig BA, Shamliyan T, Wang S-Y, Tuttle TM, et al (2010) The impact of surgery, radiation, and systemic treatment on outcomes in patients with ductal carcinoma in situ. J Natl Cancer Inst Monogr 2010:130–133 10.1093/jncimonographs/lgq022 20956816 PMC5161061

[4] Rosai J (1991) Borderline epithelial lesions of the breast. Am J Surg Pathol 15:209–221 10.1097/00000478-199103000-00001 1847606

[5] Schnitt SJ, Connolly JL, Tavassoli FA, Fechner RE, Kempson RL, et al (1992) Interobserver reproducibility in the diagnosis of ductal proliferative breast lesions using standardized criteria. Am J Surg Pathol 16:1133–1143 10.1097/00000478-199212000-00001 1463092

[6] Mommers EC, Poulin N, Sangulin J, Meijer CJ, Baak JP, et al (2001) Nuclear cytometric changes in breast carcinogenesis. J Pathol 193:33–39. 11169513 10.1002/1096-9896(2000)9999:9999<::AID-PATH744>3.0.CO;2-Q

[7] Mommers EC, Poulin N, Meijer CJ, Baak JP, van Diest PJ (2000) Malignancy-associated changes in breast tissue detected by image cytometry. Anal Cell Pathol 20:187–195. 11205321 10.1155/2000/965613 PMC4618828

[8] MacGrogan G, Arnould L, de Mascarel I, Vincent-Salomon A, Penault-Llorca F, et al (2008) Impact of immunohistochemical markers, CK5/6 and E-cadherin on diagnostic agreement in non-invasive proliferative breast lesions. Histopathology 52:689–697 10.1111/j.1365-2559.2008.03016.x 18397281

[9] Jain RK, Mehta R, Dimitrov R, Larsson LG, Musto PM, et al (2011) Atypical ductal hyperplasia: interobserver and intraobserver variability. Mod Pathol 24:917–923 10.1038/modpathol.2011.66 21532546

[10] Geller BM, Nelson HD, Carney PA, Weaver DL, Onega T, et al (2014) Second opinion in breast pathology: policy, practice and perception. J Clin Pathol 67:955–960 Available: http://www.ncbi.nlm.nih.gov/pubmed/25053542. Accessed 3 November 2014. 25053542 10.1136/jclinpath-2014-202290 PMC4521120

[11] Ghaznavi F, Evans A, Madabhushi A, Feldman M (2013) Digital imaging in pathology: whole-slide imaging and beyond. Annu Rev Pathol 8:331–359 Available: http://www.ncbi.nlm.nih.gov/pubmed/23157334. Accessed 14 June 2013. 23157334 10.1146/annurev-pathol-011811-120902

[12] Irshad H, Veillard A, Roux L, Racoceanu D (2014) Methods for nuclei detection, segmentation, and classification in digital histopathology: a review-current status and future potential. IEEE Rev Biomed Eng 7:97–114 Available: http://ieeexplore.ieee.org/articleDetails.jsp?arnumber=6690201. Accessed 10 October 2014. 24802905 10.1109/RBME.2013.2295804

[13] Gurcan MN, Boucheron LE, Can A, Madabhushi A, Rajpoot NM, et al (2009) Histopathological image analysis: a review. IEEE Rev Biomed Eng 2:147–171 10.1109/RBME.2009.2034865 20671804 PMC2910932

[14] Kothari S, Phan JH, Stokes TH, Wang MD (2013) Pathology imaging informatics for quantitative analysis of whole-slide images. J Am Med Inform Assoc 20:1099–1108 10.1136/amiajnl-2012-001540 23959844 PMC3822114

[15] Irshad H, Gouaillard A, Roux L, Racoceanu D (2014) Multispectral band selection and spatial characterization: Application to mitosis detection in breast cancer histopathology. Comput Med Imaging Graph 38:390–402 Available: http://www.sciencedirect.com/science/article/pii/S0895611114000433. Accessed 10 October 2014. 24831181 10.1016/j.compmedimag.2014.04.003

[16] Irshad H (2013) Automated mitosis detection in histopathology using morphological and multi-channel statistics features. J Pathol Inform 4:10 Available: http://www.pubmedcentral.nih.gov/articlerender.fcgi?artid=3709420&tool=pmcentrez&rendertype=abstract. Accessed 10 October 2014. 23858385 10.4103/2153-3539.112695 PMC3709420

[17] Van Diest PJ, Risse EK, Schipper NW, Baak JP, Mouriquand J (1989) Comparison of light microscopic grading and morphometric features in cytological breast cancer specimens. Pathol Res Pract 185:612–616 10.1016/S0344-0338(89)80204-3 2626371

[18] Pienta KJ, Coffey DS (1991) Correlation of nuclear morphometry with progression of breast cancer. Cancer 68:2012–2016. 1655233 10.1002/1097-0142(19911101)68:9<2012::aid-cncr2820680928>3.0.co;2-c

[19] Tan PH, Goh BB, Chiang G, Bay BH (2001) Correlation of nuclear morphometry with pathologic parameters in ductal carcinoma in situ of the breast. Mod Pathol 14:937–941 10.1038/modpathol.3880415 11598161

[20] Tahlan A, Nijhawan R, Joshi K (2000) Grading of ductal breast carcinoma by cytomorphology and image morphometry with histologic correlation. Anal Quant Cytol Histol 22:193–198. 10872034

[21] Chiusa L, Margaria E, Pich A (2000) Nuclear morphometry in male breast carcinoma: association with cell proliferative activity, oncogene expression, DNA content and prognosis. Int J Cancer 89:494–499. 11102893 10.1002/1097-0215(20001120)89:6<494::aid-ijc5>3.0.co;2-l

[22] Baak JP, Van Dop H, Kurver PH, Hermans J (1985) The value of morphometry to classic prognosticators in breast cancer. Cancer 56:374–382. 4005802 10.1002/1097-0142(19850715)56:2<374::aid-cncr2820560229>3.0.co;2-9

[23] Kronqvist P, Kuopio T, Collan Y (1998) Morphometric grading of invasive ductal breast cancer. I. Thresholds for nuclear grade. Br J Cancer 78:800–805. 9743304 10.1038/bjc.1998.582 PMC2062958

[24] Veta M, Kornegoor R, Huisman A, Verschuur-Maes AHJ, Viergever MA, et al (2012) Prognostic value of automatically extracted nuclear morphometric features in whole slide images of male breast cancer. Mod Pathol 25:1559–1565 10.1038/modpathol.2012.126 22899294

[25] Mommers EC, Page DL, Dupont WD, Schuyler P, Leonhart AM, et al (2001) Prognostic value of morphometry in patients with normal breast tissue or usual ductal hyperplasia of the breast. Int J Cancer 95:282–285. 11494225 10.1002/1097-0215(20010920)95:5<282::aid-ijc1048>3.0.co;2-x

[26] Cui Y, Koop EA, van Diest PJ, Kandel RA, Rohan TE (2007) Nuclear morphometric features in benign breast tissue and risk of subsequent breast cancer. Breast Cancer Res Treat 104:103–107 10.1007/s10549-006-9396-4 17061043 PMC2092407

[27] Dundar MM, Badve S, Bilgin G, Raykar V, Jain R, et al (2011) Computerized classification of intraductal breast lesions using histopathological images. IEEE Trans Biomed Eng 58:1977–1984 10.1109/TBME.2011.2110648 21296703 PMC3328096

[28] Pantanowitz L, Sinard JH, Henricks WH, Fatheree LA, Carter AB, et al (2013) Validating whole slide imaging for diagnostic purposes in pathology: guideline from the College of American Pathologists Pathology and Laboratory Quality Center. Arch Pathol Lab Med 137:1710–1722 10.5858/arpa.2013-0093-CP 23634907 PMC7240346

[29] Huisman A, Looijen A, van den Brink SM, van Diest PJ (2010) Creation of a fully digital pathology slide archive by high-volume tissue slide scanning. Hum Pathol 41:751–757 Available: http://www.ncbi.nlm.nih.gov/pubmed/20129646. Accessed 3 November 2014. 20129646 10.1016/j.humpath.2009.08.026

[30] Schindelin J, Arganda-Carreras I, Frise E, Kaynig V, Longair M, et al (2012) Fiji: an open-source platform for biological-image analysis. Nat Methods 9:676–682 Available: http://www.ncbi.nlm.nih.gov/pubmed/22743772. Accessed 18 October 2013. 22743772 10.1038/nmeth.2019 PMC3855844

[31] Ruifrok A, Johnston D (2001) Quantification of Histochemical Staining by Color Deconvolutions. Anal Quant Cytol Histol 23:291–299. 11531144

[32] Chang H, Loss LA, Parvin B (2012) Nuclear segmentation in H and E sections via multi-reference graph-cut (MRGC). 9th IEEE International Symposium Biomedical Imaging pp 614–617.

[33] Haralick RM, Shanmugam K, Dinstein I (1973) Textural Features for Image Classification. IEEE Trans Syst Man Cybern 3:610–621 doi 10.1109/TSMC.1973.4309314.

[34] Galloway MM (19675) Texture Analysis using Gray Level Run Lengths. Comput Graph Image Process 4:172–179.

[35] Tibshirani R (1996) Regression shrinkage and selection via the lasso. J R Statis Soc B 58:267–288.

[36] Friedman J, Hastie T, Tibshirani R (2010) Regularization Paths for Generalized Linear Models via Coordinate Descent. J Stat Softw 33:1–22 10.1359/JBMR.0301229 20808728 PMC2929880

[37] Dormann CF, Elith J, Bacher S, Buchmann C, Carl G, et al (2013) Collinearity: a review of methods to deal with it and a simulation study evaluating their performance. Ecography (Cop) 36:27–46.

[38] Beck AH, Sangoi AR, Leung S, Marinelli RJ, Nielsen TO, et al (2011) Systematic analysis of breast cancer morphology uncovers stromal features associated with survival. Sci Transl Med 3:108ra113 Available: http://www.ncbi.nlm.nih.gov/pubmed/22072638. Accessed 3 June 2013. 10.1126/scitranslmed.3002564 22072638

[39] Schultz NA, Werner J, Willenbrock H, Roslind A, Giese N, et al (2012) MicroRNA expression profiles associated with pancreatic adenocarcinoma and ampullary adenocarcinoma. Mod Pathol 25:1609–1622 10.1038/modpathol.2012.122 22878649

[40] Pellagatti A, Benner A, Mills KI, Cazzola M, Giagounidis A, et al (2013) Identification of gene expression-based prognostic markers in the hematopoietic stem cells of patients with myelodysplastic syndromes. J Clin Oncol 31:3557–3564 10.1200/JCO.2012.45.5626 24002510

[41] Razavian AS, Azizpour H, Sullivan J, Carlsson S (2014) CNN Features off-the-shelf: an Astounding Baseline for Recognition. Available: http://arxiv.org/abs/1403.6382. Accessed 3 November 2014.

[42] Cireşan DC, Giusti A, Gambardella LM, Schmidhuber J (2013) Mitosis detection in breast cancer histology images with deep neural networks. Med Image Comput Comput Assist Interv 16:411–418 Available: http://www.ncbi.nlm.nih.gov/pubmed/24579167. Accessed 3 November 2014. 24579167 10.1007/978-3-642-40763-5_51

[43] Otterbach F, Bànkfalvi A, Bergner S, Decker T, Krech R, et al (2000) Cytokeratin 5/6 immunohistochemistry assists the differential diagnosis of atypical proliferations of the breast. Histopathology 37:232–240 Available: http://www.ncbi.nlm.nih.gov/pubmed/10971699. Accessed 3 November 2014. 10971699 10.1046/j.1365-2559.2000.00882.x

